# Response to and recovery from treatment in human liver-mimetic clinostat spheroids: a model for assessing repeated-dose drug toxicity

**DOI:** 10.1093/toxres/tfaa033

**Published:** 2020-06-12

**Authors:** Stephen J Fey, Barbara Korzeniowska, Krzysztof Wrzesinski

**Affiliations:** CelVivo ApS, Middelfartvej 469, DK-5491 Blommenslyst, Denmark

**Keywords:** drug toxicity, HepG2–C3A, recovery from treatment, repeated-dose, clinostat, 3D cell culture, acetaminophen, amiodarone, diclofenac, metformin, phenformin, valproic acid

## Abstract

Medicines are usually prescribed for repeated use over shorter or longer times. Unfortunately, repeated-dose animal toxicity studies do not correlate well with observations in man. As emphasized by the ‘3Rs’ and the desire to phase-out animal research, *in vitro* models are needed. One potential approach uses clinostat-cultured 3D HepG2–C3A liver-mimetic spheroids. They take 18 days to recover *in vivo* physiological functionality and reach a metabolic equilibrium, which is thereafter stable for a year. Acute and chronic repeated-dose studies of six drugs (amiodarone, diclofenac, metformin, phenformin, paracetamol and valproic acid) suggest that spheroids are more predictive of human *in vivo* toxicity than either 2D-cultured HepG2 cells or primary human hepatocytes. Repeated non-lethal treatment results in a clear response and return to equilibrium. Mitochondrial toxic compounds can be identified using a galactose-based medium. Some drugs induced a protective (or stress) response that intensifies after the second treatment. This 3D spheroid model is inexpensive, highly reproducible and well-suited for the determination of repeated-dose toxicity of compounds (naturally or chemically synthesized).

## Introduction

Most prescription drugs are administered repeatedly either for a limited duration (for an acute illness) or for an extended period of time (for a chronic condition). Repeated-dose toxicity studies are therefore needed to characterize their toxicological profiles. The standard approach is to test in at least two species—one rodent (rats or mice) and one non-rodent (rabbits, dogs, minipigs and non-human primates) with multiples of the intended human therapeutic dose for at least 2 weeks [1, 2]. Studies are widely recognized to be expensive and difficult to translate into predicted clinical outcomes [3–6]. False positive and false negative rates, with respect to human adverse effects, may be as high as 50% [7]. Within these rates, there is variation depending on the organ affected. The highest rates of overall concordance are seen in human haematological, gastrointestinal and cardiovascular toxicities, and the lowest are cutaneous and hepatological [7]. Differences in species, statistical issues, extrapolation from high to low doses and other difficulties all compound the weaknesses of animal-based safety testing [8].

Efforts to implement the ‘3Rs’ (Reduction, Replacement and Refinement), using *in silico* or *in vitro* assays to replace animal testing for risk assessment are underway [9]. The United States Environment Protection Agency has announced a deadline of 2035 by which animal research should be eliminated [10]. This lends emphasis to develop novel *in vitro* systems for repeated-dose toxicity. *In vitro* systems using tumour-derived cell lines have often been criticized because the cells can carry many thousands of deviations from their native tissues [11] but their actual value depends on their relevance to man.

The requirements for an *in vitro* system are that it should be: viable for extended periods of time; return to the pretreated state once the drug is metabolized or removed and should reflect toxicity seen in man (an ‘*in vitro* 3Rs’: Respond, Recover and Relevant). Next-generation 3D cell culture of spheroids and organoids is widely expected to improve the effectiveness of drug toxicological predictions [12–15].

In this paper, we describe an *in vitro* 3D spheroid system that can be used for determining repeated-dose toxicity and show its utility for six commonly used drugs. It has the clear advantage that it is inexpensive compared with animal studies and, because it is based on a widely available immortal cell line, is highly comparable between different laboratories.

Amiodarone, diclofenac, metformin, phenformin and paracetamol were selected based on their diversity of structure, target organ, biological half-life and cytochrome 450 enzyme that metabolizes them. A sixth, valproic acid (VPA), has been studied previously [16]. Their important properties are presented in Table 1.

**Table 1:** Characteristics of the drugs used in this study

Drug and molecular formula	Application	Daily dose, mg/day	Toxic dose, μg/ml in blood	Biological half-life (h)
Amiodarone C_25_H_30_ClI_2_NO_3_	Cardiac dysrhythmias	Di: 800-1600 Dm: 200-600	2.5-3	30–120
Diclofenac C_14_H_10_Cl_2_NNaO_2_	Non-steroidal anti-inflammatory	50–200	50–60	1–2
Metformin C_4_H_11_N_5_	Hypoglycaemic	Di: 1000 Dm: 2000	5–10	2–4
Paracetamol C_8_H_9_NO_2_	Analgesic, anti-inflammatory, antipyretic	2–4	100–150	2–4
Phenformin C_10_H_15_N_5_	Hypoglycaemic	24–37.5	0.6	4–13
VPA C_8_H_16_O_2_	Anticonvulsant (e.g. mania, bipolar disorder)	800–3200	40–100	10–20

Di, initial dose; Dm, maintenance dose.

Amiodarone: the anti-arrhythmic mechanism of action is based on a blockage of potassium rectifier currents that are in control of cardiac repolarization occurring during Phase 3 of the cardiac action potential. Consequently, increases in action potential duration and the effective refractory period occur in cardiac myocytes leading to their reduced excitability, precluding re-entry mechanisms and ectopic foci from perpetuating tachyarrhythmias [17]. In the liver, it is oxidized to its active metabolite, desethylamiodarone by cytochrome P-450s 1A2, 2C9, 2D6 and 3A4. Amiodarone was classified by the World Health Organization as the safest and effective drug required in a health system [18]. Despite this Chen *et al*. [19], for its risk to induce drug-induced liver injury (DILI), ranked amiodorone as vMost DILI. Acute toxicity from overdose is very rare and primarily involves cardiovascular side effects including hypotension, bradycardia, ventricular tachyarrhythmias and *Torsades de pointes* [17].

Diclofenac suppresses the prostaglandin production by inhibiting COX-1 and COX-2 and is also a vMost DILI compound. Its effectiveness as an anti-inflammatory agent is much higher (×3-1000) compared with other non-steroidal anti-inflammatory drugs [20, 21]. This is due to a 4-fold higher selectivity for COX-2 isoform (overproduced in response to tissue damage and inflammation) [22, 23]. Diclofenac is metabolized by cytochrome P-450 2C9 in the liver to its 5-hydroxy derivative that is then oxidized to *N*,5-dihydroxydiclofenac in a presence of NADPH cofactor. Certain allelic variants of drug metabolizing enzymes may lead to hepatotoxicity, or the consumption of NADPH and impairment of adenosine triphosphate (ATP) synthesis have been suggested to be two major reasons of drug toxicity [24, 25].

Metformin and phenformin are both guanidine derivatives and act in a similar manner. They lower the blood glucose level by inhibiting mitochondrial complex I, reducing ATP production. Metformin also stimulates the gut glucose utilization, increased glucagon-like peptide-1 secretion and modifies the intestinal microbiome [26]. Despite complementary mechanisms of action, the drugs’ metabolism and pharmacokinetics are different. Metformin has a half-life of 2–4 h, does not bind with human plasma proteins, is metabolized by CYP3A, 2C; and 2E1 or eliminated intact from the body [27, 28]. Phenformin, has a longer half-life (7 h), is metabolized by CYP2D6 in the liver to 4-hydroxy-phenformin, which is then subsequently conjugated with glucuronic acid [29]. The toxicity of these drugs is directly connected to their mechanism of action. A shift towards anaerobic metabolism caused by drug-induced mitochondrial dysfunction leads to lactic acidosis. Phenformin was removed from the market during the 1970s because of severe toxicity [30, 31]. In contrast, metformin is widely used as a first line of treatment.

Paracetamol (or acetaminophen, APAP) exerts its analgesic and anti-inflammatory effects through inhibition of the nitric oxide pathway. In the liver, paracetamol undergoes oxidation by cytochromes 1A2 and 2E1 to *N*-acetyl-*p*-benzoquinoneimine (NAPQI) and by reacting rapidly with thiols, in particular glutathione, is converted to its glutathione adduct. Glutathione level imbalance and/or reaction of NAPQI with hepatic proteins are the main causes of toxicity [32].

## Materials and Methods

### Standard 2D cell culture conditions

The immortal human hepatocyte cell line, HepG2–C3A (ATCC CRL-10741, third passage after receipt from ATCC, Manassas, VA), were thawed from liquid nitrogen storage and cultured in standard tissue culture conditions in a customized Dulbecco’s modified Eagle’s medium (D-MEM) growth medium [87.5% D-MEM (1 g glucose/l) (Gibco, Carlsbad, CA, Cat. no. 31885-023); 1% non-essential amino acids (Gibco, Cat. no. 11140-035); 10% foetal calf serum (Sigma, St. Louis, Cat. no. F 7524); 0.5% Penicillin/Streptomycin (Gibco, Cat. no. 15140-122); 1% GlutaMAX (Gibco, Cat. no. 35050-038); 37°C, 5% CO_2_ 95% air]. To generate cells for spheroid construction, the C3A cells were initially propagated using traditional 2D tissue culture. Cells were trypsinized (0.05% trypsin/ethylenediamminetetraacetic acid, Cat. no., Gibco 15400-054) for 3 min and sown out into falcon flasks/microtitre plates and cultivated at 37°C, 5% CO_2_ 95% air in a humidified incubator. To eliminate any effects of storage in liquid nitrogen or thawing, cells were grown for at least three passages before starting the experiments, exchanging the medium every 2–3 days. Cells were used between Passages 4 and 15. The doubling time for C3A cells grown under these conditions was 76 h [33, 34].

### 3D spheroid culture conditions

#### Preparation of spheroids using AggreWell^TM^ plates

The C3A cell spheroids have been prepared with use of AggreWell™ 400 plates (Stemcel Technologies, Grenoble, France, Cat. no. 27845). Each Aggrewell™ plate has six wells and each of these contains ~4700 microwells measuring 400 μm in diameter. These plates are used to form cell aggregates. Before use, the plates were washed twice with growth medium (customized D-MEM). In order to remove all residual air bubbles from the well surface, the plates were prefilled with 0.5 ml of growth medium and centrifuged (3 min at 3000 × *g*). Cells (1.2 × 10^6^) were added to each well and the plates were centrifuged (3 min at 100 × *g*) and left in the AggreWell™ plate overnight to form spheroids.

#### Spheroid culture in bioreactors

The spheroids were detached from the AggreWell™ plates by gently washing the wells with prewarmed growth medium. The detached spheroids were collected into a Petri dish and the quality of the spheroids checked by microscopy. Compact spheroids were introduced into bioreactors (MC2 Therapeutics, Hørsholm, Denmark, Cat. no. 010). These bioreactors are specially constructed to be easy to open and close and designed to maintain 100% humidity around the growth chamber. The spheroids (~300 per bioreactor) were then cultivated at 37°C, 5% CO_2_ 95% air in a non-humidified incubator for a minimum of 21 days, exchanging the medium every 2–3 days [35].

The day when the cells were transferred into the bioreactor is defined as Day 0. An estimated 90% of the medium was changed on Day 1 and thereafter three times a week during the recovery period. To achieve a stable suspension of spheroids, the rotation speed of the bioreactors was initially set between 21 and 23 rpm and it was adjusted to compensate for the growth in size of the spheroids (and reached 27–30 rpm on Day 21). Speed adjustments were made so that inter-spheroid contact was minimized and these adjustments were made more frequently at the beginning of the culture than at the end. Because the spheroids get larger with time, the population density of the spheroids was regulated by opening the bioreactor and ‘splitting the population’ or removing excess spheroids (this usually needed to be performed once between Day 11 and 14). Before use, the spheroid batch quality was assessed by staining for 3 min. With 0.4% trypan blue (Gibco, Cat. no. 15250-061). Batches showing >90% viability were accepted.

#### Microscopy and planimetry

Photomicrographs were taken using an Olympus IX81 motorized microscope and an Olympus DP71 camera. Images were transferred to the Olympus AnalySiS^®^ Docu program (Soft Imaging System) and the ‘shadow’ area of spheroids measured using the ‘fitted polygon area’ function, which calculates the planar surface of the spheroids in μm^2^.

#### Drug treatment

Amiodarone, diclofenac, metformin, paracetamol and phenformin were purchased from Sigma (Cat nos. PHR1164-1G, PHR1144-1G, PHR1084-500MG, A7085-100G and P7045-1G, respectively). Stock solutions were prepared just before use by dissolving the compound in either growth medium or dimethyl sulfoxide (DMSO, Sigma, Cat. no. D2650) and diluted into growth medium. A control medium was prepared, which contained the same concentration of DMSO vehicle without any compound. If used, the maximum final DMSO concentration was 0.01%.

Three hundred 21-day old spheroids (corresponding to ~25 million cells or 3.5 mg protein) were gently pipetted (using a cut-tip) into each 10 ml bioreactor before treatment to provide ample material for analysis. To initiate drug treatment, the bioreactor rotation was stopped for 30 s to allow the spheroids to settle to the bottom of the bioreactor. Ninety percent of the medium volume was exchanged with medium containing the compound at concentrations that gave the final treatment concentrations (as stated in the figures). Experiments to determine the 50% lethal dose (LD_50_) were carried out initially using a broad range of drug concentrations (e.g. varying by a factor of 10) followed by two experiments using a narrow concentration range (varying typically by a factor of 2). The data from the broad range of concentrations were used to identify the final narrow range used, and so these data are only presented for phenformin. Each narrow range experiment was carried out in duplicate and ATP was measured in technical triplicates for each sample. Medium was typically exchanged at 48, 96, 168, 216 and 264 h after the start of the experiment. The spheroids were treated at Time 0 and at times indicated in the figures by the black arrows, typically when the medium was exchanged. Data were normalized to the vehicle-treated control. Error bars in the figures show standard deviation.

#### Protein determination

Previous studies have shown that the shadow area of the spheroid is closely correlated to its protein content [36]. As this is non-invasive, rapid and reliable, it was used throughout and data were expressed as a function of the amount of protein present.

#### Glucose determination

Samples for glucose determination were collected from the cultures used for the wide range phenformin dose treatments at: 0, 24, 48, 52 and 72 h and frozen until needed. Glucose was measured using a ‘Onetouch Vita’ glucose meter and test strips (MediqDanmark, Cat. nos. 64-07-078 and 64-07-079, respectively). The instrument was calibrated using a 1 mg/ml D-glucose standard solution (Sigma-Aldrich, Cat. no. G3285-5ML) and Onetouch Vita control solution (MediqDanmark, Cat. no. 64-07-081). Standards and samples were warmed to room temperature and shaken on a rotary shaker at 450 rpm for 1 h. A test strip was inserted into the instrument and 2–3 μl of each sample were spotted onto it for each measurement. The glucose concentration was read immediately. The experiment was repeated twice and data averaged. The error bars show standard deviation.

#### ATP assay

Cell viability was measured based on their ability to produce ATP [37]. Samples of the hepatocytes grown as spheroids were collected (usually 2–6 spheroids per assay point depending on the size of the spheroids) at appropriate times and transferred to white opaque microtitre plates (Nunc, Roskilde, Denmark, Cat. no. 165306), the volume of growth medium was adjusted to 100 μl. The cells were then lysed with 100 μl of lysis buffer (CellTiter-Glo luminescent cell viability assay, Promega, Fitchburg, WI, Cat. no. G7571) and shaken in the dark for 20 min. Before the luminescence was measured in a FluoStar OPTIMA (BMG Labtech, Ortenberg, Germany) using the following parameters: one kinetic window, 10 measurement cycles with 0.3 s of measurement interval time, 2-s delay per measurement, additional 0.5-s delay per position change (repeated twice for each measured plate). Replicates were performed as described under the ‘Drug treatment’ Section. The data were normalized with reference to a standard curve for ATP and to the untreated control.

## Results and Discussion

**Figure 1:**
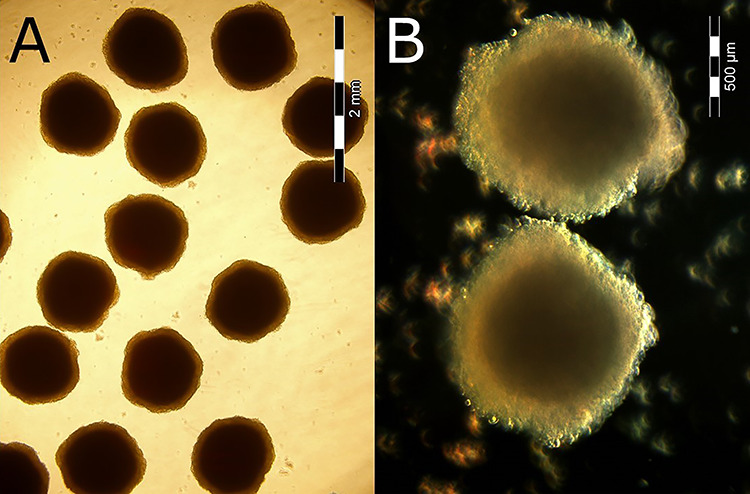
Spheroids used for the determination of the LD_50_ of medicinal compounds shown using (**A**) bright-field illumination to illustrate the uniformity of the spheroids and (**B**) dark-field illumination at higher magnification to reveal some structural details (scales were inserted by the microscope).

Previously we have shown that spheroids cultivated in an omnidirectional normogravity (often mistakenly called ‘microgravity’) clinostat bioreactor. C3A spheroids take 18 days to recover (Fig. 1) and thereafter mimic *in vivo* physiology. They maintain a metabolic equilibrium (as measured by both gene and protein expression) for at least 24 days and can be maintained for at least 300 days [38, 39]. The goal of this project was to investigate whether these spheroids were suitable for the determination of the repeated-dose LD_50_ over the course of several days. Doses were given with fresh medium at 48- or 72-h intervals (by which time most of the drug should have been metabolized, Table 1).

Spheroids formed using Aggrewell™ plates and grown in bioreactors are uniform in size (±21% Fig. 1) [36] and respond more homogeneously than spheroids of different sizes. The ‘shadow area’ of the spheroid accurately represents its protein content. This non-destructive assay allows the protein content of the actual living spheroids to be measured before the assay and used to normalize the dose given (using the lookup table presented in Supplementary Table S1). Thus, doses were given as mg compound per mg soluble protein content of the spheroids used.

The LD_50_ was measured rather than the 50% lethal concentration to facilitate comparing data between labs [36]. As a first step towards establishing the toxicity for any compound (either natural or chemically synthesized), we determined the compound’s approximate LD_50_ by treating 21-day-old C3A spheroids with doses varying by orders of magnitude. Data were normalized to the vehicle-treated control.

There are several ways to assay cell viability. For practical reasons in the preliminary ‘toxic-dose’–finding study, we determined glucose consumption from the media because this is rapid and inexpensive.

**Figure 2:**
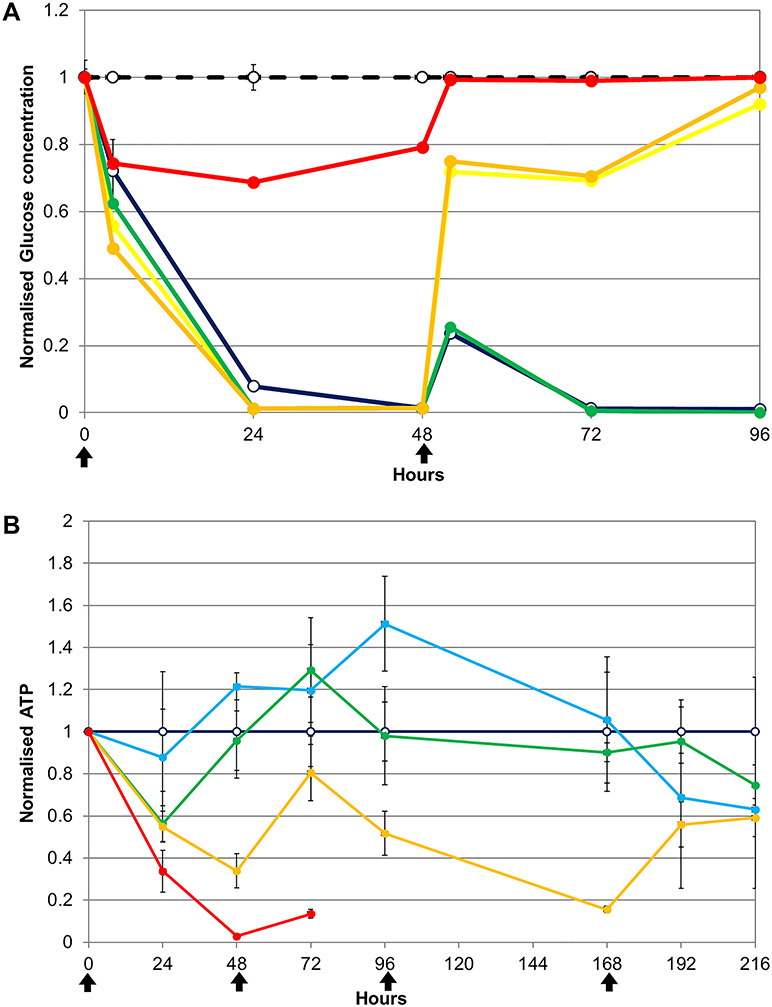
(**A**) Glucose removal from the growth medium during treatment with a wide range of phenformin doses: 0 (dark blue), 0.01 (green), 0.1 (yellow), 1 (orange), 10 mg (red) phenformin per mg soluble cellular protein present in the spheroids (‘mg/mg’) given together with the medium changes and (**B**) ATP content (viability) of spheroids treated with different doses of phenformin. 0 (dark blue, hollow marker), 0.01 (cyan), 0.02 (green), 0.04 (yellow), 0.08 mg (red) phenformin per mg protein, given together with the medium (black arrows).

Two parallel ‘mother’ spheroid cultures were each divided on Day 21 into six subcultures and exposed to phenformin at the final concentrations of 0, 0.01, 0.1, 1, 10 mg phenformin per mg soluble protein at Time 0 and 48 h. The ‘0’ concentration control spheroid culture was treated with the vehicle (final DMSO concentration, 0.1%). A further bioreactor, maintained without cells, was also treated with the vehicle to exclude the possibility that the drug caused a chemical breakdown of the glucose. Data were averaged and normalized to the amount of glucose present in the cell-free cultures. Spheroids treated with the highest dose (10 mg phenformin per mg protein, red line) consumed some of the glucose from the medium within the first 3 h but thereafter were not able to metabolize anymore (Fig. 2A). Following the 48-h medium change (and second phenformin dose), these spheroids were dead.

Spheroids treated with 0, 0.01, 0.1 and 1 mg/mg phenformin all rapidly consumed glucose for the first 48 h. All phenformin-treated spheroids metabolized the glucose faster than the untreated spheroids, illustrating that the drug treatment stimulated their metabolism. This is probably a defensive ‘stress’ response [16]. Following the medium change and second phenformin treatment, the two spheroid cultures with intermediate doses of 0.1 and 1 mg/mg managed to metabolize some of the glucose in the first 3 h but were thereafter dead. The lowest dose and the control spheroids were able to rapidly metabolize the glucose throughout the experiment. The LD_50_ for phenformin under these growth conditions was between 0.1 and 0.01. Similar wide range dose-finding experiments were carried out for all compounds tested (not shown) as a prelude to the narrow range studies presented here.

Measurement of ATP is one of the best indicators of cellular viability [37]. Therefore, ATP production was used for LD_50_ determination using narrow dose range of phenformin concentrations (0.01, 0.02, 0.04 and 0.08 mg/mg) during 9 days. The highest phenformin dose (0.08 mg/mg) was acutely toxic and one dose killed the cells (Fig. 2B). The 0.04 mg/mg dose caused a significant reduction in ATP production after the first dose but did not kill the cells. After the 48 h, the cells still produced 81% of the ATP. This capacity fell with time and if the experiment had been prolonged >9 days, the spheroids would probably have died. The lower doses (0.02 and 0.01 mg/mg) were clearly insufficient to kill the cells and at times resulted in an increased ATP production. Thus, the acute lethal phenformin dose was determined to be 0.8 mg/mg and the chronic lethal dose was 0.4 mg/mg.

**Figure 3:**
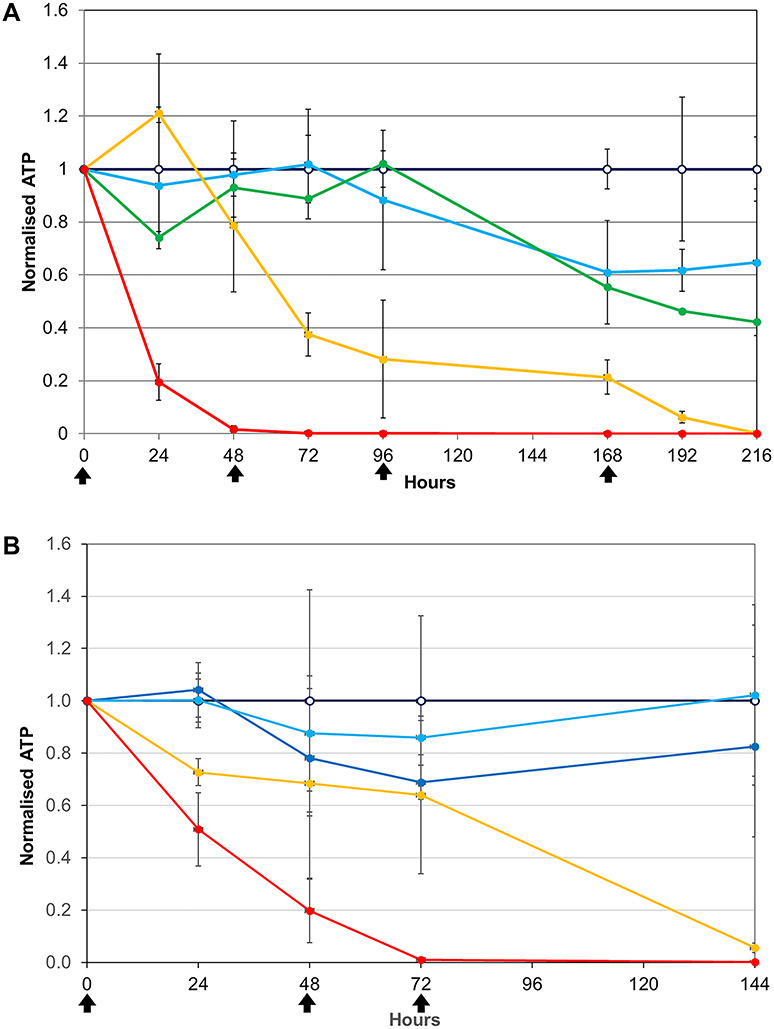
(**A**) ATP content (viability) of spheroids treated with different doses of diclofenac: 0 (dark blue, hollow marker), 0.0375 (cyan) 0.75 (green), 1.5 (yellow), 3.0 mg/mg (red) and (**B**) ATP content (viability) of spheroids treated with different doses of amiodarone: 0 (dark blue, hollow marker), 0.1 (light blue) 0.2 (cyan), 0.4 (yellow) and 0.8 mg/mg (red).

Diclofenac and amiodarone give essentially similar results to those seen for phenformin except that they did not induce a significant increase in ATP (Fig. 3A and B). The acute and chronic lethal doses for diclofenac were 3 and 1 mg/mg, respectively, and for amiodarone were 0.9 and 0.4 mg/mg.

Some drugs are toxic to mitochondria. However, cells can produce ATP via aerobic glycolysis as well as by oxidative phosphorylation and this could result in the cells surviving even though the tricarboxylic cycle is inhibited. As particular tissues, spheroids and cancers are more reliant on aerobic glycolysis than oxidative phosphorylation (Warburg effect) [33, 34], this would make spheroids less susceptible to mito-toxic drugs.

Replacing the glucose energy source with galactose can identify mito-toxic compounds. Pyruvate production via glycolytic metabolism of glucose yields two net ATP molecules. In contrast, pyruvate production via glycolytic metabolism of galactose yields no net ATP. Cells thus become reliant on oxidative phosphorylation for energy [40, 41].

**Figure 4:**
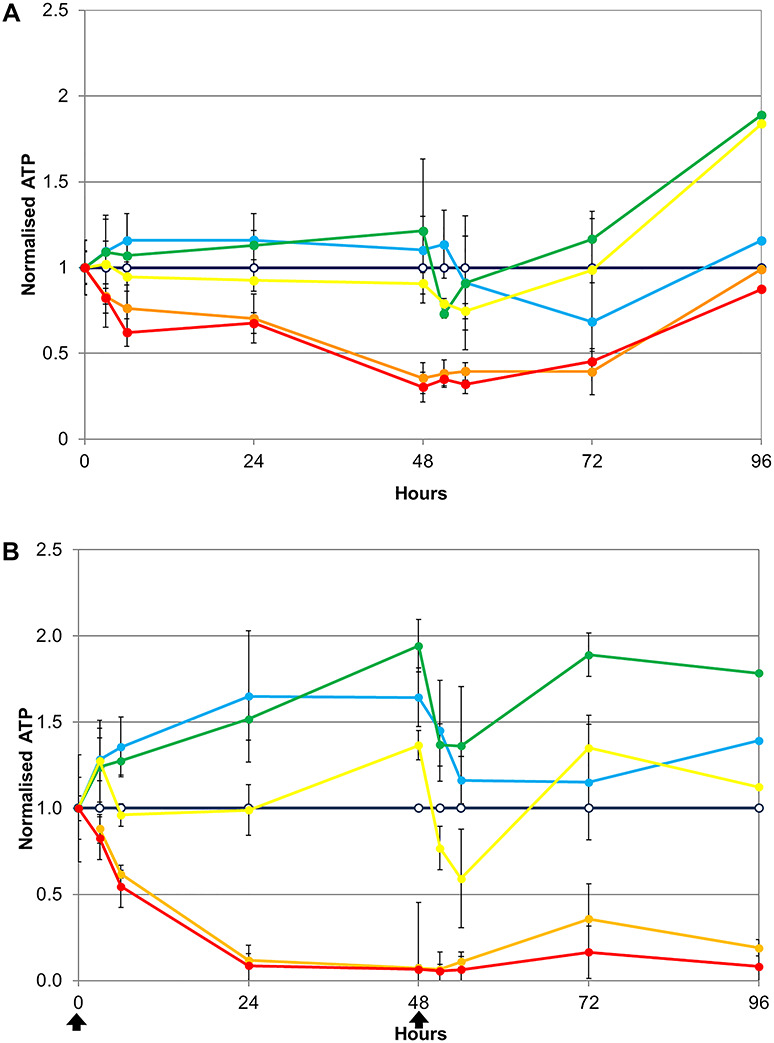
(**A**) ATP content (viability) of spheroids grown in a glucose containing medium and treated with metformin at doses 0 (dark blue, hollow marker), 0.05 (cyan), 0.5 (green), 1.0 (yellow), 2.5 (orange) and 5.0 mg/mg (red) and (**B**) ATP content (viability) of spheroids grown in a galactose containing medium and treated with the same doses of metformin.

Metformin selectively and weakly inhibits the mitochondrial respiratory chain complex 1 and decrease nicotinamide adenine dinucleotide oxidation [42, 43]. Metformin was therefore investigated, cultivating spheroids in glucose and galactose media (Fig. 4A and B). When glucose was available, even the highest concentration of metformin used (5 mg/mg) ‘only’ produced a 70% reduction in ATP production (Fig. 4A). At 96 h (after the second dose), ATP production recovered. In stark contrast, in galactose medium, both the highest and the second highest metformin doses resulted in a sustained, >90% inhibition of ATP production (Fig. 4B). Lower doses caused increased ATP production, characteristic of the defence mechanism noted above.

Additional samples were taken at 3 and 6 h after changing the medium (i.e. at 51 and 54 h). These revealed that, at all of the non-lethal doses, the spheroids responded to the second metformin treatment and then were able to recover to equilibrium.

Amiodarone has been suggested to cause liver injury due to inhibition of the mitochondrial respiratory chain complexes I and II [44–46]. Amiodarone treatment of spheroids with the galactose medium showed no change in the LD_50_ suggesting that the primary cytotoxic effect is not on oxidative phosphorylation (not shown).

**Figure 5:**
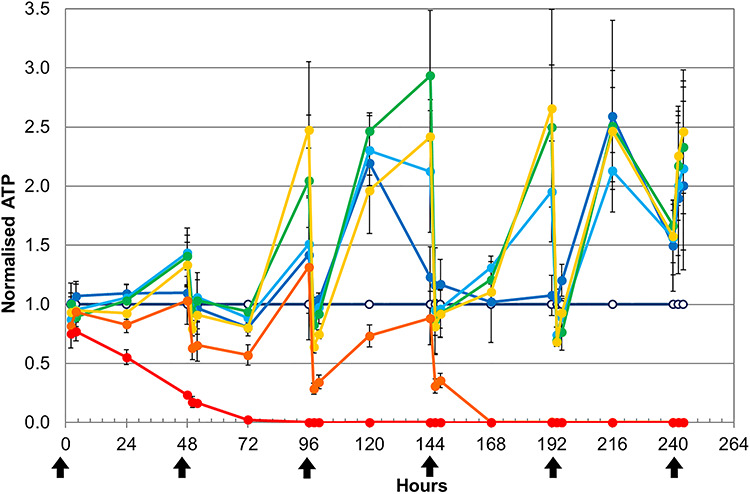
ATP content (viability) of spheroids grown in a glucose containing medium and treated with paracetamol at the following doses: 0 (dark blue, hollow marker), 0.625 (blue), 1.25 (cyan), 2.5 (green), 5 (yellow), 10 (orange) and 20 mg/mg (red).

In order to investigate the spheroids’ response to drug treatment in detail, spheroids were treated six times with various doses of paracetamol at 48-h intervals during a 10-day period (Fig. 5). Samples were collected at the time the medium was exchanged and in addition, 3 and 6 h thereafter.

In total, 20 mg paracetamol per mg protein was shown to be acutely lethal. Half of this dose was found to be chronically lethal, where four treatments killed the spheroids.

Further 2-fold dose reductions stimulated ATP production. The initial fall in ATP levels was followed by a ‘defensive’ stimulation (as seen for phenformin, Fig. 2B, and metformin, Fig. 4B).

Interestingly, at the lower doses of 5, 2.5 1.25 and 0.625 mg/mg, the stimulation of the ATP response did not appear to be dose dependent. One possible explanation is that these cells have reached a maximum tolerable ATP limit.

The response to all doses became stronger after the first two treatments, suggesting that the spheroids were adapting. They may be producing additional glutathione and reducing rates of protein synthesis and degradation [47].

Both of these features are significant because multiple doses are relevant for patient treatment. The therapeutic dose is 4 g/day [47, 48] resulting in a blood concentration of 10–25 μg/ml [49, 50]. If the ATP levels are indicators of the therapeutic effect, then treatment is maximized already at very low doses. Thus, a lower therapeutic dose might be sufficient and maintenance doses could be even lower.

In a similar study of VPA, the acute and chronic toxicity thresholds were determined to be 20 and 4 mg VPA per mg protein, respectively [16]. This ratio is comparable to those ratios determined here.

### Consequence for the perceived therapeutic index

The therapeutic index (or ratio) is the ratio between therapeutic and lethally toxic doses (normally determined). Averaged for >180 widely used medicinal compounds, the therapeutic index is only 12.5 [50]. The results presented here indicate that the average ratio between the acute and chronic thresholds for lethal toxicity is about three. Thus, if the medicine is prescribed for repeated dosing, the average therapeutic index may be only about 4-fold.

### Biological half-life of the compound

*In vitro*, compounds or their metabolites are not removed as they would be *in vivo* and thus their *in vitro* half-lives may be longer. In the model described here, treatment was repeated each 48 or 72 h. Exchange of 90% of the spent medium will partially mimic removal by the kidneys. As the biological half-life of most of the compounds is shorter, this might be too infrequent. However, 48-h treatment intervals minimize disturbances to the spheroid cultures.

**Figure 6:**
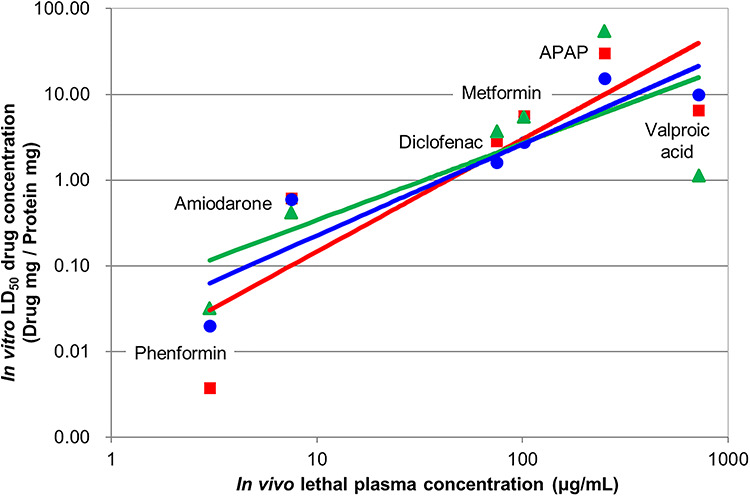
Correlation for six drugs between the LD_50_ observed in three different *in vitro* models against *in vivo* lethal dose observed in man: Green triangles and line indicate aggregated published data from 2D-cultured HepG2 cell lines, *R*^2^ = 0.547; red squares and line indicate data from primary human hepatocytes, *R*^2^ = 0.747; and blue circles and line: indicate aggregated published data from 3D-cultured HepG2–C3A spheroids, *R*^2^ = 0.854.

### Predictiveness of *in vitro* data of *in vivo* toxicity

One important question that remains to be answered is how predictive the *in vitro* toxicity determination is compared with that observed *in vivo* (i.e. persons who accidentally or deliberately were exposed to lethal doses). This question was previously approached by averaging all the literature available for five compounds (phenformin, amiodarone, metformin, paracetamol and VPA) [36]. The data were divided into three categories: (i) 2D cultures of HepG2– and HepG2–C3A; (ii) primary human hepatocytes and (iii) 3D cultures of HepG2–C3A. All the *in vitro* toxicity data were converted to LD_50_ (mg compound per mg protein) and compared with the *in vivo* comatose or lethal dose [50].

Here we add data for diclofenac. Intramuscular injection of either 75 or 100 mg diclofenac resulted in death for two patients [51, 52]. Assuming a total blood volume of 5 l and that all of the diclofenac was dissolved, the lethal blood dose of diclofenac would correspond to 15–20 μg/ml. This is low compared with the published data of a non-lethal toxic dose of 50–60 μg/ml [50, 53, 54]. However, intramuscular injection could lead to a lethal bolus of diclofenac. Therefore, for the purposes of comparing *in vitro* and *in vivo* doses here, a ‘conservative’ lethal dose of 75 μg/ml was used. Variation in this estimate between 50 and 100 made little difference to the correlation obtained.

Returning to the comparison of predictive value of toxicity determinations *in vitro* and *in vivo*, HepG2 when grown in the classical 2D cell culture results in a poor correlation (Fig. 6, green line, *R*^2^ = 0.547). Fresh primary human hepatocytes (the pharmaceutical industry’s ‘gold’ standard) resulted in a better correlation (red line, *R*^2^ = 0.747). HepG2–C3A 3D spheroids resulted in the best correlation (blue line, *R*^2^ = 0.854).

It should be noted that the 2D-cultured HepG2 and primary human hepatocyte data were collected using different assays in different labs. These data showed noticeable variation, and so the averages used here should be treated with caution [36].

## Conclusions

Firstly, C3A spheroids can provide a practical model for the determination of lethal toxicity thresholds *in vitro* (both acute and chronic). Their ratio suggests that the average therapeutic index is 3-fold lower than commonly assumed. This might have significance for patients who repetitively take medicine. Secondly, some subtoxic drug concentrations activate ATP production. This is assumed to be a protective (or stress) response. Thirdly, subtoxic drug treatment of spheroids results in a clear response and then a return to the ‘metabolic equilibrium’ from which the cells were displaced. For the same dose, the response of the cells following subsequent treatments may not be the same as the initial response. Fourthly, galactose medium can be used to identify mito-toxic compounds. Fifthly, toxicity determinations made using C3A spheroids are at least as predictive as primary human hepatocytes of *in vivo* toxicity. Finally, because of the simplicity of the clinostat bioreactor system, it is possible to test repeated drug treatments over extended periods of time. The longest treatment presented here was six treatments in 10 days but could have been extended. Thus, C3A spheroids offer an inexpensive, highly reproducible alternative, which would be comparable between labs. Therefore, C3A spheroids can be used for repeated-dose screening of candidate drugs (whether natural compounds or synthetic).

## Funding

MC2 Therapeutics, Hørsholm, Denmark, the University of Southern Denmark and CelVivo ApS have all sponsored this work.

## Conflict of Interest Statement

The funders had no role in study design, data collection and analysis, decision to publish or preparation of the manuscript. The authors are employed by CelVivo ApS and have used equipment sold by CelVivo as part of their research.

## Author Contributions

Conceptualization and evaluation were carried out by S.J.F. and K.W.; methodology was prepared by K.W.; investigation was performed by S.J.F., B.K. and K.W.; writing was completed by S.J.F., B.K. and K.W.; visualization was performed by S.J.F.; administration and funding acquisition was carried out by S.J.F and K.W.

## Supplementary Material

200316_ToxRes_Supplementary_mat_Table_1_tfaa033

## References

[1] Peters TS Do preclinical testing strategies help predict human hepatotoxic potentials? Toxicol Pathol 2005;33:146–54. 15805066 10.1080/01926230590522121

[2] Van Summeren A, Renes J, Delft JH van et al. Proteomics in the search for mechanisms and biomarkers of drug-induced hepatotoxicity. Toxicol In Vitro 2012;26:373–85. 22274661 10.1016/j.tiv.2012.01.012

[3] Waring MJ, Arrowsmith J, Leach AR et al. An analysis of the attrition of drug candidates from four major pharmaceutical companies. Nat Rev Drug Discov 2015;14:475–86. 26091267 10.1038/nrd4609

[4] Griesinger C, Desprez B, Coecke S et al. Validation of alternative in vitro methods to animal testing: concepts, challenges, processes and tools. Adv Exp Med Biol 2016;856:65–132. 27671720 10.1007/978-3-319-33826-2_4

[5] McGonigle P, Ruggeri B Animal models of human disease: challenges in enabling translation. Biochem Pharmacol 2014;87:162–71. 23954708 10.1016/j.bcp.2013.08.006

[6] Lynch S, Pridgeon CS, Duckworth CA et al. Stem cell models as an in vitro model for predictive toxicology. Biochem J 2019;476:1149–58. 30988136 10.1042/BCJ20170780 PMC6463389

[7] Olson H, Betton G, Robinson D et al. Concordance of the toxicity of pharmaceuticals in humans and in animals. Regul Toxicol Pharmacol 2000;32:56–67. 11029269 10.1006/rtph.2000.1399

[8] Basketter DA, Clewell H, Kimber I et al. A roadmap for the development of alternative (non-animal) methods for systemic toxicity testing. ALTEX 2012;29:3–91. 22307314 10.14573/altex.2012.1.003

[9] Jaroch K, Jaroch A, Bojko B Cell cultures in drug discovery and development: the need of reliable in vitro-in vivo extrapolation for pharmacodynamics and pharmacokinetics assessment. J Pharm Biomed Anal 2018;147:297–312. 28811111 10.1016/j.jpba.2017.07.023

[10] Grimm D U.S. EPA to eliminate all mammal testing by 2035. Science 2019. doi:10.1126/science.aaz4593

[11] Goodspeed A, Heiser LM, Gray JW et al. Tumor-derived cell lines as molecular models of cancer pharmacogenomics. Mol Cancer Res 2016;14:3–13. 26248648 10.1158/1541-7786.MCR-15-0189 PMC4828339

[12] Astashkina A, Grainger DW Critical analysis of 3-D organoid in vitro cell culture models for high-throughput drug candidate toxicity assessments. Adv Drug Deliv Rev 2014;69–70:1–18. 10.1016/j.addr.2014.02.008 24613390

[13] Turnaturi R, Chiechio S, Salerno L et al. Progress in the development of more effective and safer analgesics for pain management. Eur J Med Chem 2019;183:111701. 31550662 10.1016/j.ejmech.2019.111701

[14] Molero P, Ramos-Quiroga JA, Martin-Santos R et al. Antidepressant efficacy and tolerability of ketamine and esketamine: a critical review. CNS Drugs 2018;32:411–20. 29736744 10.1007/s40263-018-0519-3

[15] Rosenholm JM, Mamaeva V, Sahlgren C et al. Nanoparticles in targeted cancer therapy: mesoporous silica nanoparticles entering preclinical development stage. Nanomedicine (Lond) 2012;7:111–20. 22191780 10.2217/nnm.11.166

[16] Fey SJ Wrzesinski K Determination of acute lethal and chronic lethal dose thresholds of valproic acid using 3D spheroids constructed from the immortal human hepatocyte cell line HepG2/C3A In: Valproic Acid: Pharmacology, Mechanisms of Action and Clinical Implications. New York: Nova Science Publishers, 2013, Ch. V, 141–165.

[17] Florek JB, Girzadas D Treasure Island. Florida: StatPearls, 2020.

[18] World Health Organization Model List of Essential Medicines, 21st List. Geneva: World Health Organization, 2019.

[19] Chen M, Suzuki A, Thakkar S et al. DILIrank: the largest reference drug list ranked by the risk for developing drug-induced liver injury in humans. Drug Discov Today 2016;21:648–53. 26948801 10.1016/j.drudis.2016.02.015

[20] Ku EC, Lee W, Kothari HV et al. The effects of diclofenac sodium on arachidonic acid metabolism. Semin Arthritis Rheum 1985;15:36–41. 3936178 10.1016/s0049-0172(85)80008-1

[21] Ku EC, Lee W, Kothari HV et al. Effect of diclofenac sodium on the arachidonic acid cascade. Am J Med 1986;80:18–23. 10.1016/0002-9343(86)90074-4 3085488

[22] Tegeder I, Pfeilschifter J, Geisslinger G Cyclooxygenase-independent actions of cyclooxygenase inhibitors. FASEB J 2001;15:2057–72. 11641233 10.1096/fj.01-0390rev

[23] Vane JR, Botting RM Mechanism of action of anti-inflammatory drugs. Scand J Rheumatol Suppl 1996;102:9–21. 8628981 10.3109/03009749609097226

[24] National Institute of Diabetes and Digestive and Kidney Diseases LiverTox: Clinical and Research Information on Drug-Induced Liver Injury 2012. Bethesda, MD: National Institute of Diabetes and Digestive and Kidney Diseases, 2017. 31643176

[25] Bort R, Ponsoda X, Jover R et al. Diclofenac toxicity to hepatocytes: a role for drug metabolism in cell toxicity. J Pharmacol Exp Ther 1998;288:65–72. 9862754

[26] Rena G, Hardie DG, Pearson ER The mechanisms of action of metformin. Diabetologia 2017;60:1577–85. 28776086 10.1007/s00125-017-4342-z PMC5552828

[27] Bell PM, Hadden DR Metformin. Endocrinol Metab Clin North Am 1997;26:523–37. 9314013 10.1016/s0889-8529(05)70265-6

[28] Tucker GT, Casey C, Phillips PJ et al. Metformin kinetics in healthy subjects and in patients with diabetes mellitus. Br J Clin Pharmacol 1981;12:235–46. 7306436 10.1111/j.1365-2125.1981.tb01206.x PMC1401849

[29] Shah RR, Evans DA, Oates NS et al. The genetic control of phenformin 4-hydroxylation. J Med Genet 1985;22:361–6. 4078865 10.1136/jmg.22.5.361 PMC1049479

[30] Bailey CJ Biguanides and NIDDM. Diabetes Care 1992;15:755–72. 1600835 10.2337/diacare.15.6.755

[31] Bridges HR, Jones AJ, Pollak MN et al. Effects of metformin and other biguanides on oxidative phosphorylation in mitochondria. Biochem J 2014;462:475–87. 25017630 10.1042/BJ20140620 PMC4148174

[32] Graham GG, Davies MJ, Day RO et al. The modern pharmacology of paracetamol: therapeutic actions, mechanism of action, metabolism, toxicity and recent pharmacological findings. Inflammopharmacology 2013;21:201–32. 23719833 10.1007/s10787-013-0172-x

[33] Wrzesinski K, Rogowska-Wrzesinska A, Kanlaya R et al. The cultural divide: exponential growth in classical 2D and metabolic equilibrium in 3D environments. PLoS One 2014;9:e106973. 25222612 10.1371/journal.pone.0106973 PMC4164521

[34] Wrzesinski K, Fey SJ Metabolic reprogramming and the recovery of physiological functionality in 3D cultures in micro-bioreactors. Bioengineering (Basel) 2018;5:22. 10.3390/bioengineering5010022 PMC5874888 29518979

[35] Wrzesinski K Molecular Markers Associated with Hepatotoxicity: Development of In Vitro Test System Based on Human Cells. Saarbrücken, Germany: Vdm, 2009.

[36] Fey SJ, Wrzesinski K Determination of drug toxicity using 3D spheroids constructed from an immortal human hepatocyte cell line. Toxicol Sci 2012;127:403–11. 22454432 10.1093/toxsci/kfs122 PMC3355318

[37] Riss TL, Moravec RA, Niles AL Cytotoxicity testing: measuring viable cells, dead cells, and detecting mechanism of cell death. Methods Mol Biol 2011;740:103–14. 21468972 10.1007/978-1-61779-108-6_12

[38] Wrzesinski K, Fey SJ After trypsinisation, 3D spheroids of C3A hepatocytes need 18 days to re-establish similar levels of key physiological functions to those seen in the liver. Toxicol Res 2013;2:123–35.

[39] Wrzesinski K, Magnone MC, Visby Hansen L et al. HepG2/C3A 3D spheroids exhibit stable physiological functionality for at least 24 days after recovering from trypsinisation. Toxicol Res 2013;2:163–72.

[40] Aguer C, Gambarotta D, Mailloux RJ et al. Galactose enhances oxidative metabolism and reveals mitochondrial dysfunction in human primary muscle cells. PLoS One 2011;6:e28536. 22194845 10.1371/journal.pone.0028536 PMC3240634

[41] Marroquin LD, Hynes J, Dykens JA et al. Circumventing the Crabtree effect: replacing media glucose with galactose increases susceptibility of HepG2 cells to mitochondrial toxicants. Toxicol Sci 2007;97:539–47. 17361016 10.1093/toxsci/kfm052

[42] El-Mir MY, Nogueira V, Fontaine E et al. Dimethylbiguanide inhibits cell respiration via an indirect effect targeted on the respiratory chain complex I. J Biol Chem 2000;275:223–8. 10617608 10.1074/jbc.275.1.223

[43] Vial G, Detaille D, Guigas B Role of mitochondria in the mechanism(s) of action of metformin. Front Endocrinol (Lausanne) 2019;10:294. 31133988 10.3389/fendo.2019.00294 PMC6514102

[44] Fromenty B, Fisch C, Berson A et al. Dual effect of amiodarone on mitochondrial respiration. Initial protonophoric uncoupling effect followed by inhibition of the respiratory chain at the levels of complex I and complex II. J Pharmacol Exp Ther 1990;255:1377–84. 1979817

[45] Li N, Oquendo E, Capaldi RA et al. A systematic assessment of mitochondrial function identified novel signatures for drug-induced mitochondrial disruption in cells. Toxicol Sci 2014;142:261–73. 25163676 10.1093/toxsci/kfu176

[46] Papiris SA, Triantafillidou C, Kolilekas L et al. Amiodarone: review of pulmonary effects and toxicity. Drug Saf 2010;33:539–58. 20553056 10.2165/11532320-000000000-00000

[47] Wojdyla K, Wrzesinski K, Williamson J et al. Acetaminophen-induced S-nitrosylation and S-sulfenylation changes in 3D cultured hepatocarcinoma cell spheroids. Toxicol Res 2016;5:905–20. 10.1039/c5tx00469a PMC6072433 30090399

[48] Heard KJ, Green JL, James LP et al. Acetaminophen-cysteine adducts during therapeutic dosing and following overdose. BMC Gastroenterol 2011;11:20. 21401949 10.1186/1471-230X-11-20 PMC3066114

[49] Heard K, Bui A, Mlynarchek SL et al. Toxicity from repeated doses of acetaminophen in children: assessment of causality and dose in reported cases. Am J Ther 2014;21:174–83. 22407198 10.1097/MJT.0b013e3182459c53 PMC3374904

[50] Schulz M, Schmoldt A Therapeutic and toxic blood concentrations of more than 800 drugs and other xenobiotics. Pharmazie 2003;58:447–74. 12889529

[51] Alkhawajah AM, Eifawalb M, Mahmoud SF Fatal anaphylactic reaction to diclofenac. Forensic Sci Int 1993;60:107–10. 8340033 10.1016/0379-0738(93)90098-u

[52] Schabitz WR, Berger C, Knauth M et al. Hypoxic brain damage after intramuscular self-injection of diclofenac for acute back pain. Eur J Anaesthesiol 2001;18:763–5. 11580784 10.1046/j.1365-2346.2001.00913.x

[53] Davies NM, Anderson KE Clinical pharmacokinetics of diclofenac. Therapeutic insights and pitfalls. Clin Pharmacokinet 1997;33:184–213. 9314611 10.2165/00003088-199733030-00003

[54] Fowler PD, Shadforth MF, Crook PR et al. Plasma and synovial fluid concentrations of diclofenac sodium and its major hydroxylated metabolites during long-term treatment of rheumatoid arthritis. Eur J Clin Pharmacol 1983;25:389–94. 6628528 10.1007/BF01037953

