# Supplementary material for: Response to and recovery from treatment in human liver-mimetic clinostat spheroids: a model for assessing repeated-dose drug toxicity
Source: Toxicol Res (Camb). 2020 Jun 12;9(4):379–89. doi: 10.1093/toxres/tfaa033 (PMC7467243; doi:10.1093/toxres/tfaa033)
Supplement: 200316_ToxRes_Supplementary_mat_Table_1_tfaa033 [file 200316_toxres_supplementary_mat_table_1_tfaa033.pdf]

## PROTEIN CONTENT PER SPHEROID
## AS A FUNCTION OF AGE AND SHADOW AREA

| Bioreactor culture<br><br>days | Average spheroid size<br>mm² | Average protein concentration<br>µg/mm² | Protein content per spheroid<br>µg | Number of cells per spheroid<br>calc. | Protein content of 300 spheroids<br>calc. |
|---|---|---|---|---|---|
| 0 | 0.033 | 7.00 | 0.23 | 1,642 | 70 |
| 1 | 0.071 | 8.09 | 0.57 | 4,028 | 172 |
| 2 | 0.104 | 9.10 | 0.94 | 6,610 | 283 |
| 3 | 0.132 | 10.04 | 1.33 | 9,294 | 398 |
| 4 | 0.158 | 10.90 | 1.72 | 12,023 | 516 |
| 5 | 0.181 | 11.69 | 2.12 | 14,769 | 635 |
| 6 | 0.203 | 12.42 | 2.52 | 17,529 | 755 |
| 7 | 0.223 | 13.10 | 2.92 | 20,316 | 876 |
| 8 | 0.243 | 13.73 | 3.34 | 23,156 | 1,001 |
| 9 | 0.263 | 14.31 | 3.76 | 26,085 | 1,129 |
| 10 | 0.284 | 14.85 | 4.21 | 29,142 | 1,264 |
| 11 | 0.305 | 15.35 | 4.69 | 32,370 | 1,407 |
| 12 | 0.329 | 16.27 | 5.75 | 39,513 | 1,725 |
| 13 | 0.353 | 15.82 | 5.20 | 35,813 | 1,560 |
| 14 | 0.380 | 16.69 | 6.35 | 43,511 | 1,904 |
| 15 | 0.410 | 17.09 | 7.00 | 47,843 | 2,099 |
| 16 | 0.441 | 17.47 | 7.71 | 52,542 | 2,312 |
| 17 | 0.475 | 17.84 | 8.48 | 57,636 | 2,545 |
| 18 | 0.512 | 18.20 | 9.33 | 63,148 | 2,798 |
| 19 | 0.552 | 18.56 | 10.24 | 69,096 | 3,073 |
| 20 | 0.594 | 18.91 | 11.24 | 75,492 | 3,371 |
| 21 | 0.639 | 19.27 | 12.31 | 82,342 | 3,694 |
| 22 | 0.686 | 19.62 | 13.47 | 89,646 | 4,040 |
| 23 | 0.736 | 19.99 | 14.71 | 97,398 | 4,412 |
| 24 | 0.787 | 20.36 | 16.03 | 105,586 | 4,809 |
| 25 | 0.841 | 20.74 | 17.43 | 114,191 | 5,230 |
| 26 | 0.896 | 21.13 | 18.92 | 123,187 | 5,676 |
| 27 | 0.951 | 21.53 | 20.49 | 132,541 | 6,146 |
| 28 | 1.008 | 21.95 | 22.13 | 142,211 | 6,638 |
| 29 | 1.064 | 22.39 | 23.83 | 152,148 | 7,150 |
| 30 | 1.120 | 22.85 | 25.60 | 162,294 | 7,680 |
| 31 | 1.175 | 23.33 | 27.42 | 172,582 | 8,225 |
| 32 | 1.229 | 23.82 | 29.27 | 182,935 | 8,782 |
| 33 | 1.280 | 24.34 | 31.15 | 193,264 | 9,345 |
| 34 | 1.328 | 24.88 | 33.03 | 203,471 | 9,910 |
| 35 | 1.371 | 25.45 | 34.90 | 213,443 | 10,470 |
| 36 | 1.411 | 26.04 | 36.72 | 223,055 | 11,017 |
| 37 | 1.444 | 26.65 | 38.47 | 232,168 | 11,542 |
| 38 | 1.471 | 27.28 | 40.12 | 240,623 | 12,036 |
| 39 | 1.490 | 27.94 | 41.62 | 248,244 | 12,486 |
| 40 | 1.500 | 28.62 | 42.93 | 254,834 | 12,879 |
| 41 | 1.500 | 29.32 | 44.00 | 260,169 | 13,200 |
| 42 | 1.490 | 30.05 | 44.77 | 263,996 | 13,431 |

**Number of cells is calculated from the protein content**
**Data is for HepG2/C3A cells cultured in a CelVivo clinostat bioreactor**
**Values for other cell lines may vary from those given**
